# Concurrent pulmonary infection and perinephric abscess: a case report and literature review

**DOI:** 10.3389/fimmu.2025.1528542

**Published:** 2025-07-07

**Authors:** Lixin Guo, Li Ma, Wenjing Ren, Xiaoli Tang, Yuqiu Hao, Peng Gao

**Affiliations:** ^1^ Department of Respiratory Medicine, The Second Hospital of Jilin University, Changchun, Jilin, China; ^2^ Department of Surgery, Shanghai Sixth People’s Hospital Affiliated to Shanghai Jiao Tong University School of Medicine, Shanghai, China

**Keywords:** pulmonary infection, perinephric abscess, targeted next-generation sequencing, oral anaerobes, case report

## Abstract

This report details the case of a 64-year-old male with well-controlled type 2 diabetes mellitus and hypertension. The patient presented with a 20-day history of progressive dyspnea, cough, and intermittent fever, which worsened despite antibiotic treatment. The initial assessment revealed leukocytosis, neutrophilia, and abnormal chest computed tomography (CT) findings, which led to a provisional diagnosis of pulmonary infection. However, empirical antibacterial therapy was ineffective. Further investigations revealed a right perinephric abscess and empyema caused by an oral anaerobic bacterial infection. Although the sputum cultures were negative, targeted next-generation sequencing (tNGS) identified multiple oral anaerobes. The patient was treated with metronidazole and drainage. After 33 days, the symptoms and laboratory abnormalities gradually resolved. Follow-up over one year demonstrated complete resolution of symptoms, normalization of inflammatory markers and no recurrence of the infection. This case highlights the importance of considering occult anaerobic infections in refractory febrile patients with diabetes, while also raising awareness of the rare complication of perinephric abscess and highlighting the value of tNGS in pathogen identification.

## 1 Introduction

Perinephric abscess (PA), characterized by the invasion of pathogens within the perinephric space, is a relatively infrequent yet severe medical condition (1). Recent evidence has refined our understanding of its etiology, clinical manifestations, diagnosis, and management (2). Hematogenous spread from a distant site, ascending urinary tract infections, and direct extension from adjacent organs are the major causes of PA. Moreover, there is an emerging recognition of the role of underlying immunocompromised states, such as those associated with advanced malignancies or chronic immunosuppressive therapy (3, 4). Despite controversy, research has further elaborated on the impact of comorbidities such as diabetes mellitus on the development of PA (5). Patients with diabetes often have impaired immune function and hyperglycemia-induced tissue damage, creating a favorable environment for bacterial growth and abscess formation (6). *Escherichia coli* is the most commonly isolated pathogen, followed by *Staphylococcus aureus*, as demonstrated in multiple contemporary microbiological surveys (2, 7). Clinically, patients with PA present with non-specific symptoms and typically experience fever, flank pain, and malaise (2). Nevertheless, these symptoms can be easily overlooked or misinterpreted, especially in patients with preexisting comorbidities (7). Currently, diagnosis relies heavily on advanced imaging modalities. High-resolution computed tomography (CT) remains the gold standard for detecting the presence, location, and extent of the abscess (2, 8). Ultrasonography has auxiliary value in diagnosing PA, but the diagnostic utility of magnetic resonance imaging (MRI) is limited (2). PA management includes antibiotic administration with concurrent percutaneous drainage, if necessary (2, 9). Surgical intervention may be used when PA is not successfully controlled with antibiotic treatment or percutaneous catheter drainage (2). However, the choice between percutaneous drainage and surgical intervention still depends on various factors such as size, location, and complexity of the abscess, as well as the patient’s overall condition. Furthermore, the use of adjunctive therapies, such as hyperbaric oxygen therapy, has been investigated to enhance the efficacy of standard treatment by facilitating an improvement in the bactericidal properties of neutrophils, increasing the penetration of antibiotics into damaged tissues, and reducing the systemic inflammatory response, thus aiding in the repair of damaged tissues (10). When considering patients with PA who also have concurrent pulmonary infections, the clinical scenario becomes more complex. A previous study reported pleural empyema in a patient with a PA and diaphragmatic defect (11). The patient was successfully treated with systemic antibiotics and drainage of both pleural and retroperitoneal collections (11). The authors concluded that the spread of intra-abdominal sepsis through diaphragmatic defects to the pleural cavity represented a potential source of empyema (11). Thus, understanding the coexistence of pulmonary infection and PA is of the utmost importance, as it impacts the diagnostic approach, treatment strategy, and overall prognosis. Herein, we report a case of a patient presenting with concurrent pulmonary infection and PA to contribute to the growing body of literature on this complex and relatively rare clinical entity.

## 2 Case presentation

**Figure 1:**
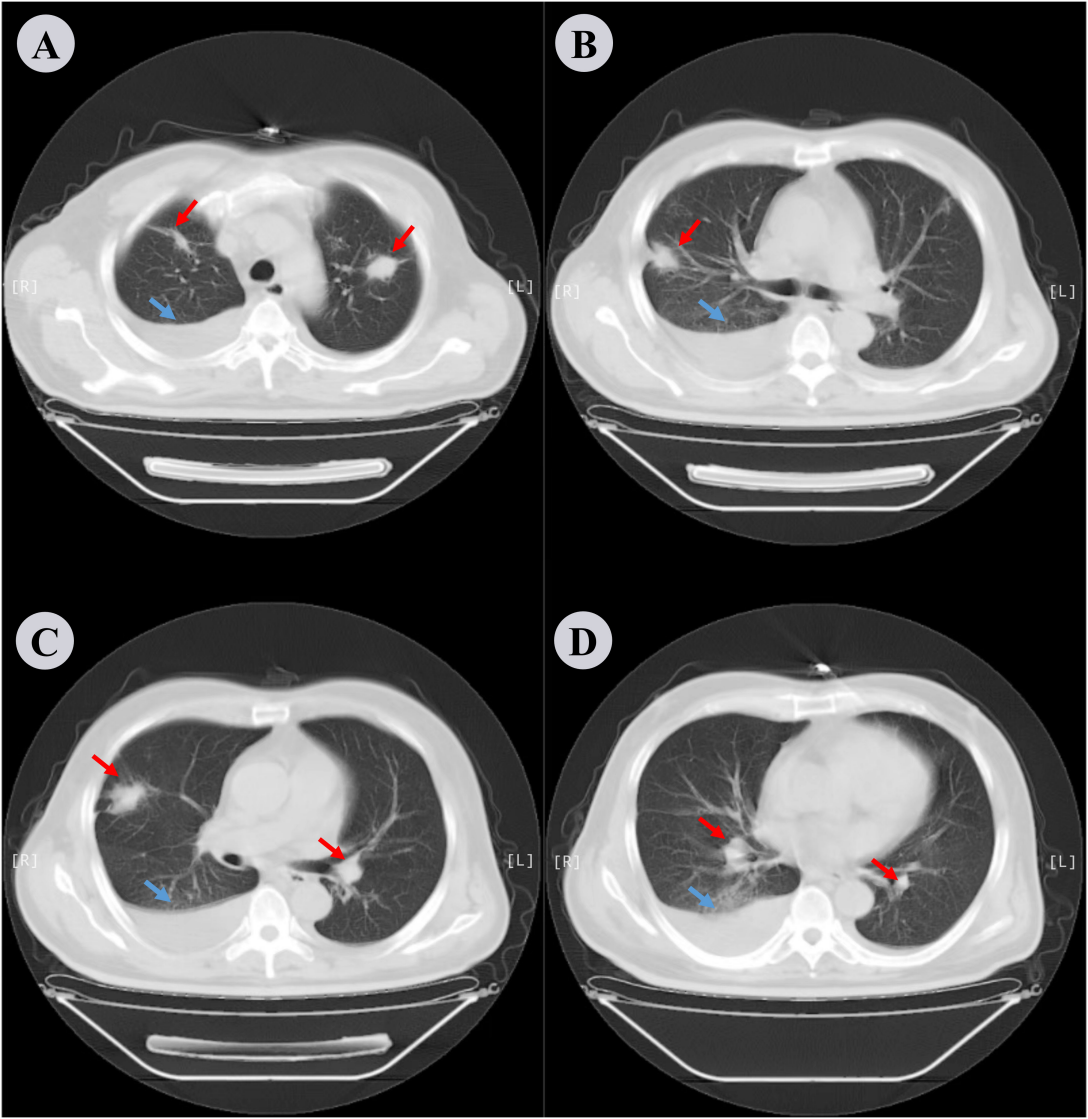
Chest CT scan performed the day before hospitalization. **(A−D)** depict different slices of the lung CT scans in the lung window. Bilateral pleural effusion is noted along with compressive atelectasis in the lower right lung and scattered chronic inflammation in both lungs. Red arrows indicate inflammation in the lungs and blue arrows indicate pleural effusion.

A 64-year-old male patient presented with a 20-day history of progressive dyspnea, cough, and intermittent fever, which worsened despite a 10-day course of cephalosporins and penicillin from a local medical institution. Initial laboratory tests revealed leukocytosis (white blood cell [WBC] count: 20.94 × 10^9^/L) with neutrophilia (87.6%). Chest CT revealed bilateral pleural effusion, compressive atelectasis in the right lower lung, and diffuse chronic inflammation (**Figure 1**). A provisional diagnosis of pulmonary infection was made, however, empiric antibacterial therapy failed to alleviate the symptoms. The patient was subsequently transferred to the respiratory department for further management. At admission, he reported persistent high-grade fever (peak: 39.5°C), chills, cough, and exertional dyspnea relieved by rest. No history of aspiration, chest pain, gastrointestinal symptoms, or weight loss was noted, and the patient denied any history of smoking or alcohol consumption. His medical history included well-controlled type 2 diabetes mellitus, hypertension, and mild obstructive sleep apnea syndrome (OSA). Vital signs included a temperature of 38.5°C, blood pressure of 151/79 mmHg, heart rate of 88/min, respiratory rate of 23/min, and oxygen saturation of 93% on low-flow oxygen. Physical examination revealed diminished breath sounds and moist rales in both lower lung fields. Repeat blood tests showed marked leukocytosis (WBC: 23.10 × 10^9^/L), with neutrophilia (90.4%) and lymphopenia (4.2%). Urinalysis results were unremarkable.

**Figure 2:**
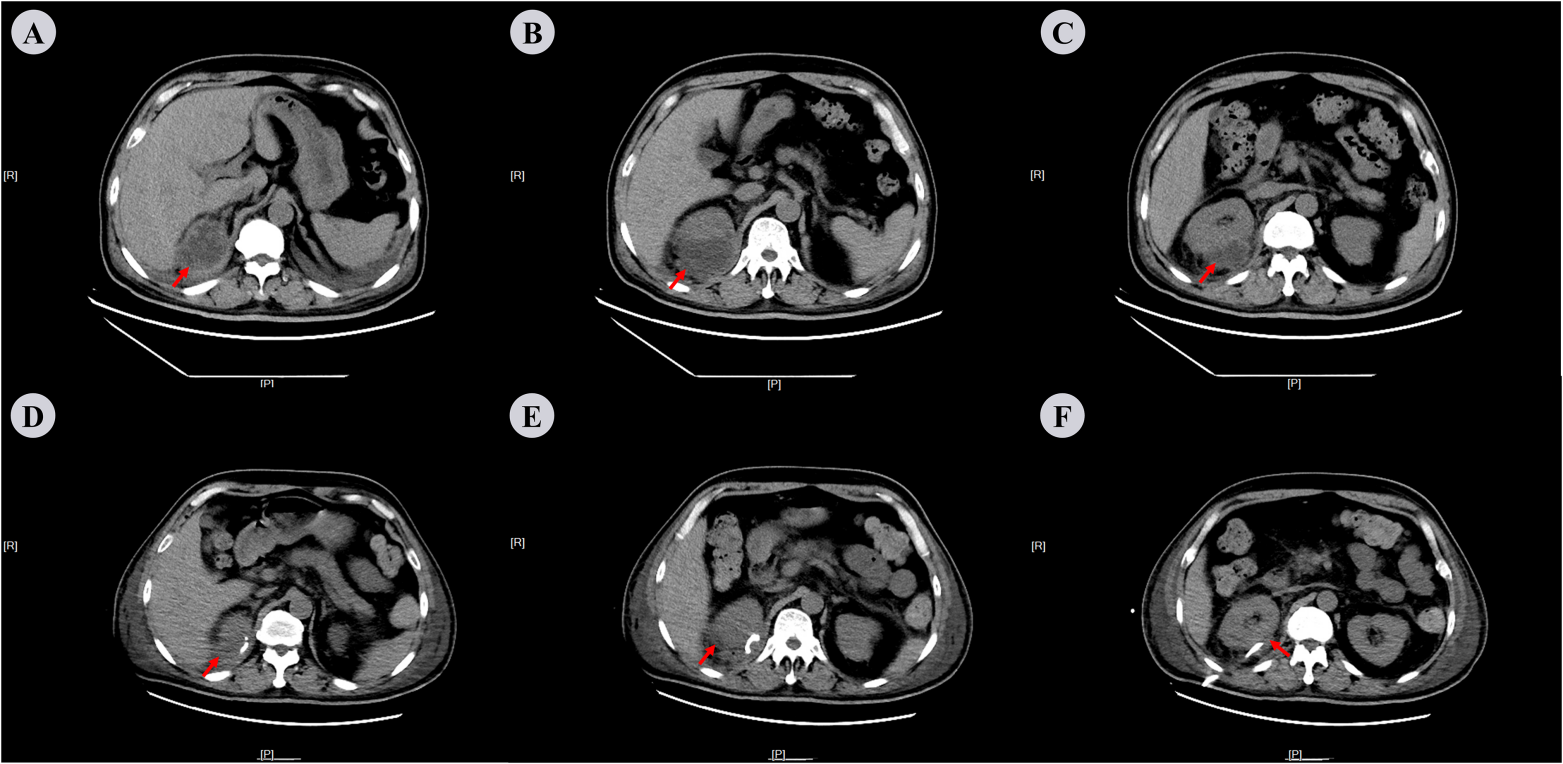
Abdominal CT scan on the 3rd and 33rd days after admission. **(A−C)** On the 3rd day: Mass-like heterogeneous density shadow with irregular margins, adjacent to the upper pole of the right kidney. The lesion shows internal septations and measures approximately 62 mm × 49 mm × 61 mm in dimensions with the CT attenuation value varying from -21 Hu to -12 Hu. **(D−F)** On the 13th day: Following the right renal drainage procedure, a small patchy low-density shadow was visible adjacent to the right kidney, with measures around 39 mm × 14 mm × 3 mm in size and a CT attenuation value of roughly 15Hu. The red arrows indicate perinephric abscess.

**Figure 3:**
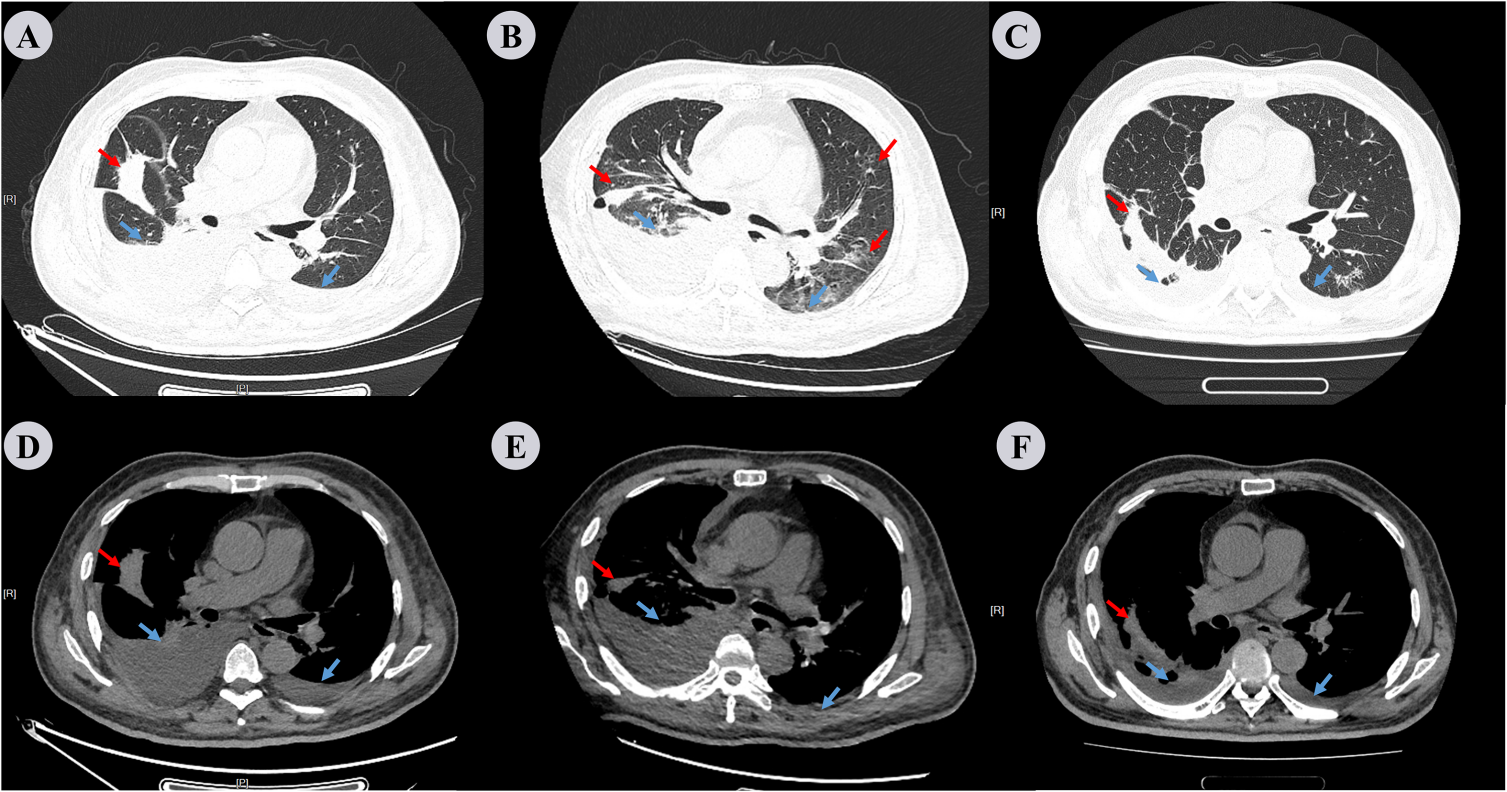
Chest CT scan performed on the 3rd, 13th, and 33rd days. **(A−C)** represent the lung window, while **(D−F)** represent the mediastinal window. Repeat chest CT on the 3rd day **(A, D)**, compared to the pre-admission scan, revealed that the lesions in both lungs had no change; however, there was an increase in the amount of pleural effusion, especially on the right side. Repeat chest CT on 13th day **(B, E)** reveals increased inflammatory lesions in both lungs and decreased pleural effusion. Repeat chest CT on the 33rd day **(C, F)** shows that the inflammation in both lungs had decreased significantly compared to the previous images, and the right pleural effusion also decreased markedly. Red arrows denote inflammation in the lungs and blue arrows indicate pleural effusion.

The patient was diagnosed with a pulmonary infection, type 2 diabetes mellitus, and grade 2 hypertension. He received insulin therapy, oxygen support, intravenous hydration, and meropenem as empirical anti-infective treatment, (1 g IV q8h). Despite treatment, fever persisted on day 3. Sputum cultures remained negative, and serum-tNGS detected only human herpesvirus. Elevated B-type natriuretic peptide (BNP: 481 pg/mL) and hypoalbuminemia (22.3 g/L) were noted. The differential diagnoses included bacteremia and occult extrapulmonary infection, prompting an abdominal CT examination. This revealed a heterogeneous mass-like lesion with internal septations adjacent to the right renal upper pole (**Figures 2A–C**). Repeat chest CT showed unchanged pulmonary infiltrates, but increased right pleural effusion (**Figures 3A, D**). Ultrasound-guided drainage of the right perirenal lesion yielded 200 mL of pink purulent fluid, confirming a PA. Pus cultures were negative; however, tNGS identified multiple oral anaerobic bacteria (**Table 1**). Two days later, the patient reported chest tightness. A bedside chest X-ray in the supine position demonstrated bilateral pleural effusion, which was more pronounced on the right side (**Supplementary Figure S1**). Concurrent bilateral thoracentesis under ultrasound guidance drained yellow fluid. The left-sided pleural effusion was close to leaking, while the right-sided effusion met the Light criteria for exudate (**Table 2**). Consistently, tNGS of the right-sided pleural fluid detected oral anaerobes (**Table 1**). Tinidazole (0.5 g IV q12h) was initiated for anaerobic coverage. Clinical improvement ensued with the resolution of fever, cough, and dyspnea. Repeat abdominal and chest CT on day 13 showed reduced PA (**Figures 2D–F**) and pleural effusion, although pulmonary inflammation persisted with right pleural septations (**Figures 3B, E**). Ultrasound-guided urokinase intrapleural injection was used to treat the right pleural septations, and the gradual resolution of symptoms and laboratory abnormalities was observed over 33 days. Follow-up chest CT revealed significant inflammatory absorption (**Figures 3C, F**).The timeline of the patient’s diagnostic workup and clinical care throughout the case is detailed in **Supplementary Figure S2**, **Supplementary Figure S3**.

**Table 1:** Species distribution of pathogens detected by right perinephric abscess and right pleural effusion tNGS.

Pathogen	Relative abundance (%)	
	Right perinephric abscess	Right pleural effusion
*Parvimonas micra*	28.79	0.71
*Streptococcus constellatus*	17.12	9.01
*Treponema denticola*	13.41	21.10
*Prevotella buccae*	3.47	0
*Porphyromonas gingivalis*	1.26	20.39
*Torque teno virus*	25.03	86.08

**Table 2:** Examination results of bilateral pleural effusion, including both routine and biochemical analyses.

Sample Type	Appearance	Rivalta	WBC-BF *10^6^/L	Multinuclear cell (%)	Monocytes (%)	Total protein (g/L)	LDH (U/L)
Right-sided hydrothorax	Deep yellow	**+**	4146	64.1	35.9	38.1	758
Left-sided hydrothorax	Yellowish	**+/-**	228	13.2	86.8	12.8	65

**Figure 4:**
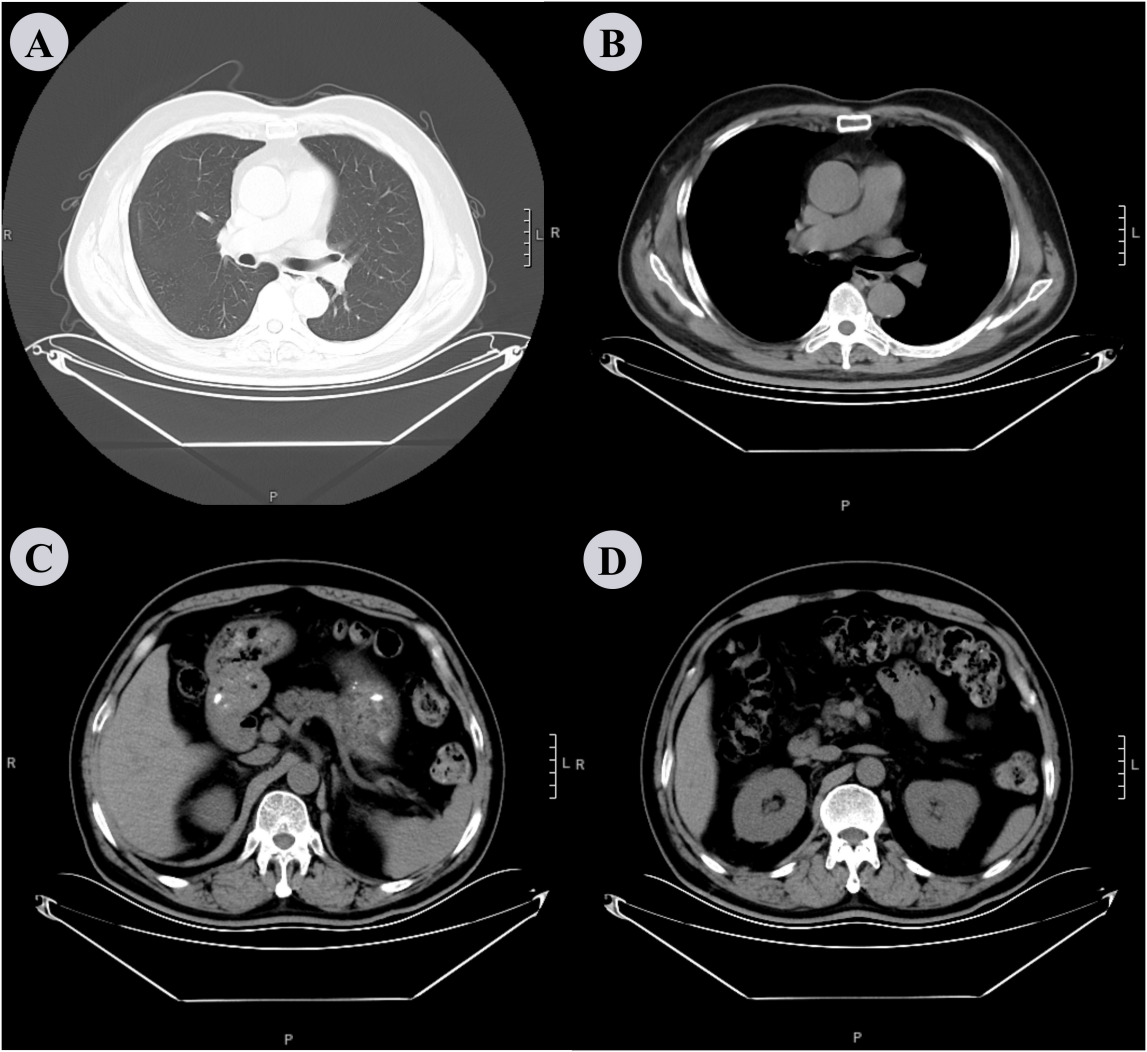
Chest and abdominal CT scan performed after 1 year. **(A, B)** The chest CT scan shows complete resolution of bilateral pleural effusion and pulmonary infiltrates, with no residual abscess or septations. **(C, D)** shows the absence of recurrent perirenal collections, although there is slight thickening of the perirenal fascia.

The patient underwent regular clinical and radiographic follow-ups for over one year after discharge. At the one-month follow-up, dyspnea, cough, and fever had completely resolved, with no recurrence of chills. Repeat laboratory tests revealed normalization of inflammatory markers (WBC: 6 × 10^9^/L, neutrophils: 51.6%). One-year follow-up chest CT revealed complete resolution of the bilateral pleural effusion and pulmonary infiltrates, with no residual abscess or septations (**Figures 4A, B**). Abdominal CT confirmed the absence of recurrent perirenal collections (**Figures 4C, D**). Functional recovery was notable as the patient resumed daily activities without exertional limitations. No antibiotic-related adverse effects (e.g., gastrointestinal disturbances or neurotoxicity) or procedural complications (e.g., catheter-site infections or pneumothorax) were observed during or after treatment. Tinidazole was discontinued after a 4-week course, with no signs of relapse at subsequent evaluations. This case underscored the importance of investigating occult anaerobic infections in patients with diabetes and refractory fever, particularly when thoracic and abdominal imaging studies reveal discordant findings. The integration of tNGS facilitated pathogen identification despite negative conventional cultures, thereby guiding targeted antimicrobial therapy.

## 3 Discussion

Previous studies have indicated that most cases of PA result from the direct spread of pyelonephritis, with gram-negative bacilli, especially *E. coli*, being the most common pathogen (51.4%) (2). PA caused by *S. aureus* is usually secondary to hematogenous seeding (2). Additionally, certain anaerobes can also cause PA, and routine bacterial cultures are typically negative (12). Anaerobic bacteria have been isolated from PA associated with previous abdominal surgeries and renal transplants (12). In the present case, the detection of multiple oral anaerobic bacteria in PA using tNGS suggested that anaerobic infections should be considered, especially when conventional cultures are negative. Notably, the patient did not undergo abdominal surgery or renal transplantation. Regarding the origin of the pulmonary infection with pleural effusion, two main possibilities exist: aspiration pneumonia and respiratory tract colonization. Aspiration pneumonia often occurs when oral or gastric contents are inhaled into the lower respiratory tract. Although the patient denied a history of aspiration, he did have a history of mild obstructive sleep apnea (OSA). Therefore, the presence of oral anaerobes in both the PA and pleural effusion could not rule out the possibility of occult aspiration. Some studies have shown that, in patients with poor oral hygiene, the load of oral anaerobes increases, and even minor aspiration events, such as microaspiration during sleep, can lead to the entry of these bacteria into the lungs, causing infection (13, 14). Conversely, respiratory tract colonization by oral anaerobes may also be a factor, as oral anaerobes can colonize the respiratory tract in patients with a compromised immune system or airway clearance function (15, 16). The repeated negative sputum cultures in this case made it difficult to determine whether the presence of oral anaerobes in the pleural effusion was due to aspiration pneumonia or colonization. It is possible that the sampling method for sputum cultures was not optimal for detecting these fastidious anaerobes, or that the organisms were present in low numbers in the sputum at the time of sampling.

Hematogenous spread is a common route by which bacteria reach the perinephric area (2). However, in this case, serum tNGS results were negative for oral anaerobes. It should be noted that the sensitivity of blood NGS for detecting low-level bacteremia is relatively low, with a high false-negative rate. Anaerobic bacteria may be present transiently or in small quantities in the bloodstream, which makes them difficult to detect (17). Direct extension is another possible route despite the absence of anatomical abnormalities in the diaphragm (11). The anatomical structure known as the lumbar rib triangle is located between the lumbar and rib regions of the diaphragm. This area lacks muscle fibers and is composed only of the supradiaphragmatic and infradiaphragmatic fasciae, making it a weak part of the diaphragm. Additionally, the lumbar rib triangle is closely related to the kidneys, especially the upper pole of the kidneys. In this patient, there was significant right-sided pleural effusion, which could cause physical pressure on the diaphragm, particularly in the area of the lumbar rib triangle, affecting its normal function. Additionally, inflammatory damage to the lungs and pleural cavity may have weakened the integrity of the diaphragm (18), providing a potential pathway for the spread of oral anaerobes from the thoracic cavity to the perinephric area. Although this route is less common, it cannot be completely excluded, especially considering the proximity of the right lung infection to the diaphragm and the fact that the perinephric abscess was also located at the upper pole of the kidney.

A comprehensive treatment approach was adopted. Meropenem was initially used as a broad-spectrum antibacterial treatment. Meropenem has a wide antibacterial spectrum and strong antibacterial activity, and can cover many common aerobic and anaerobic bacteria. However, the patient’s fever persisted, indicating that the initial treatment was ineffective. Considering the tNGS results, tinidazole was then added. Tinidazole is highly effective against anaerobic bacteria and can penetrate their cell membranes, interacting with their DNA, inhibiting DNA synthesis and leading to bacterial death (19). In addition to antibacterial therapy, drainage is also crucial. Ultrasound-guided percutaneous drainage of the perinephric abscess effectively removed the purulent fluid, reducing the bacterial load and the source of infection. Similarly, bilateral thoracentesis under ultrasound guidance drained the pleural effusion, relieving pressure on the lungs and providing samples for microbiological analyses. When right pleural septations occurred, the use of urokinase for intrapleural injection was an important adjunctive measure. Urokinase can dissolve fibrin clots and septations in the pleural cavity, thereby improving the drainage effect (11).

This case study provides several important insights. First, occult anaerobic infections should be suspected in patients with diabetes and refractory fever, especially when accompanied by abnormal chest and abdominal findings. Diabetes can compromise the immune system and increase the risk of infection by opportunistic pathogens (6). Second, the limitations of traditional culture methods in detecting anaerobic bacteria highlight the importance of advanced molecular techniques, such as tNGS, which can identify pathogens even when conventional cultures are negative, guiding targeted antimicrobial therapy (20, 21). Third, for patients with concurrent thoracic and abdominal manifestations, possible routes of bacterial spread, such as hematogenous and direct extension, should be carefully considered. Finally, long-term management should focus on optimizing glycemic control and providing nutritional support to improve overall immune function and reduce the risk of infection recurrence.

## 4 Conclusion

In conclusion, this case demonstrated the complexity of diagnosing and treating patients with diabetes and refractory fever. The identification of oral anaerobic bacteria as the causative agents through tNGS was crucial for effective treatment. The possible routes of dissemination of oral anaerobes to the perinephric area require further investigation. An integrated treatment approach, including appropriate antibiotic selection and drainage procedures, was key to the patient’s recovery. This case emphasizes the importance of considering anaerobic infections in similar patients and the value of advanced diagnostic techniques. Therefore, clinicians should be vigilant in such cases to improve the diagnosis and treatment of complex infections.

## Data Availability

The datasets presented in this study can be found in online repositories. The names of the repository/repositories and accession number(s) can be found below: https://www.cncb.ac.cn/, PRJCA038549.

## Supplementary material

Supplementary Figure 1 Chest X-ray examination. A blurring of the costophrenic angles shows the existence of substantial bilateral pleural effusion.

Supplementary Figure 2 Timeline of the patient’s diagnostic workup. This figure illustrates the key chronological events in the patient’s diagnostic and therapeutic process. The timeline includes: onset of symptoms, initial presentation, diagnostic evaluations, date of diagnosis, initiation of Treatment, follow-up visits. Arrows indicate the sequence of events.

Supplementary Figure 3 Nursing record. **(A)** Shows the patient’s temperature, heart rate, and blood oxygen level changes at key time points during the illness. **(B)** Shows the changes in pleural effusion drainage and perinephric abscess drainage at key time points during the illness.
