# Supplementary figures and images for: Concurrent pulmonary infection and perinephric abscess: a case report and literature review
Source: Front Immunol. 2025 Jul 7;16:1528542. doi: 10.3389/fimmu.2025.1528542 (PMC12277380; doi:10.3389/fimmu.2025.1528542)

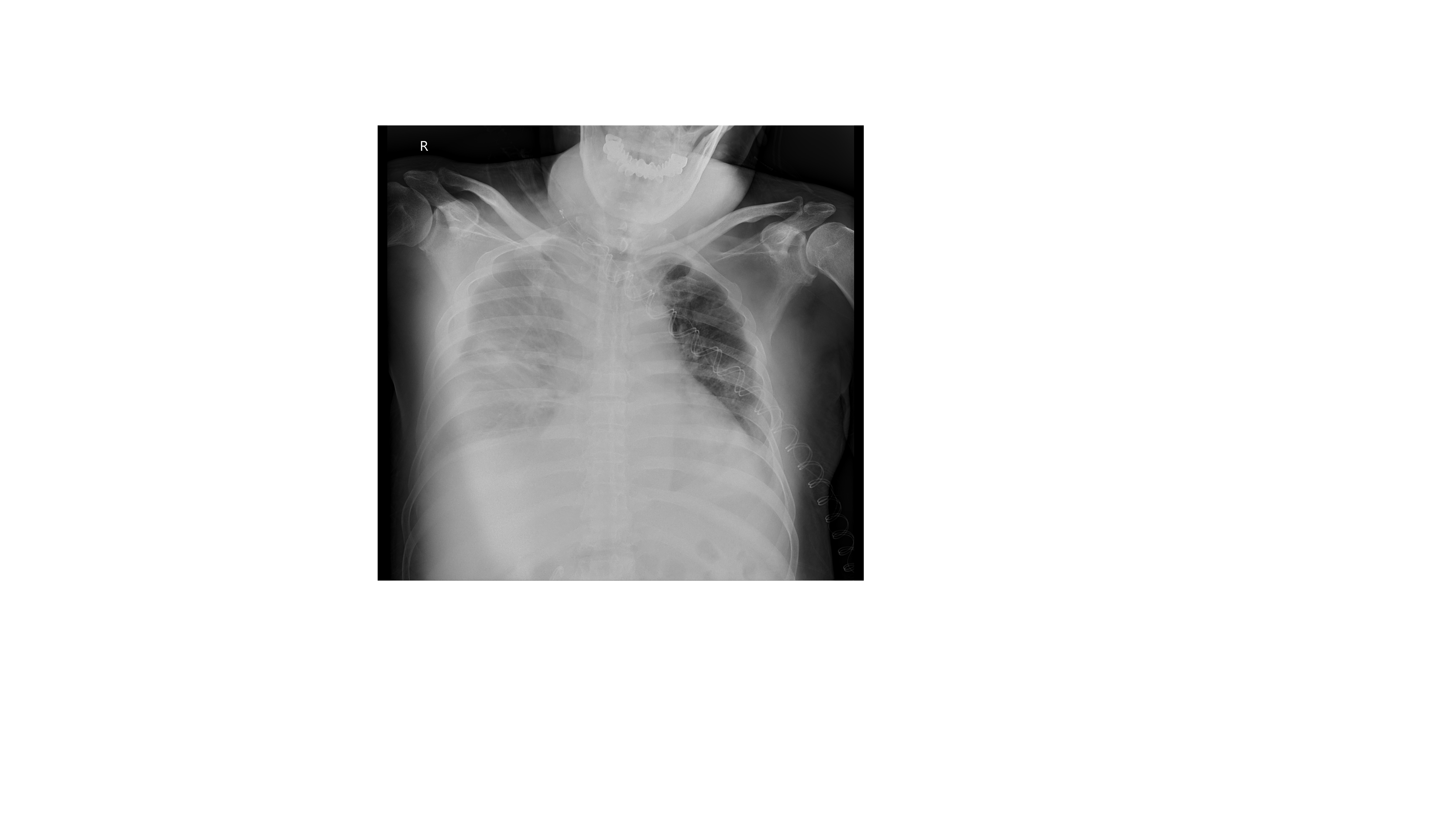

Supplement: Supplementary Figure 1 — Chest X-ray examination. A blurring of the costophrenic angles shows the existence of substantial bilateral pleural effusion. [file Image1.tif]

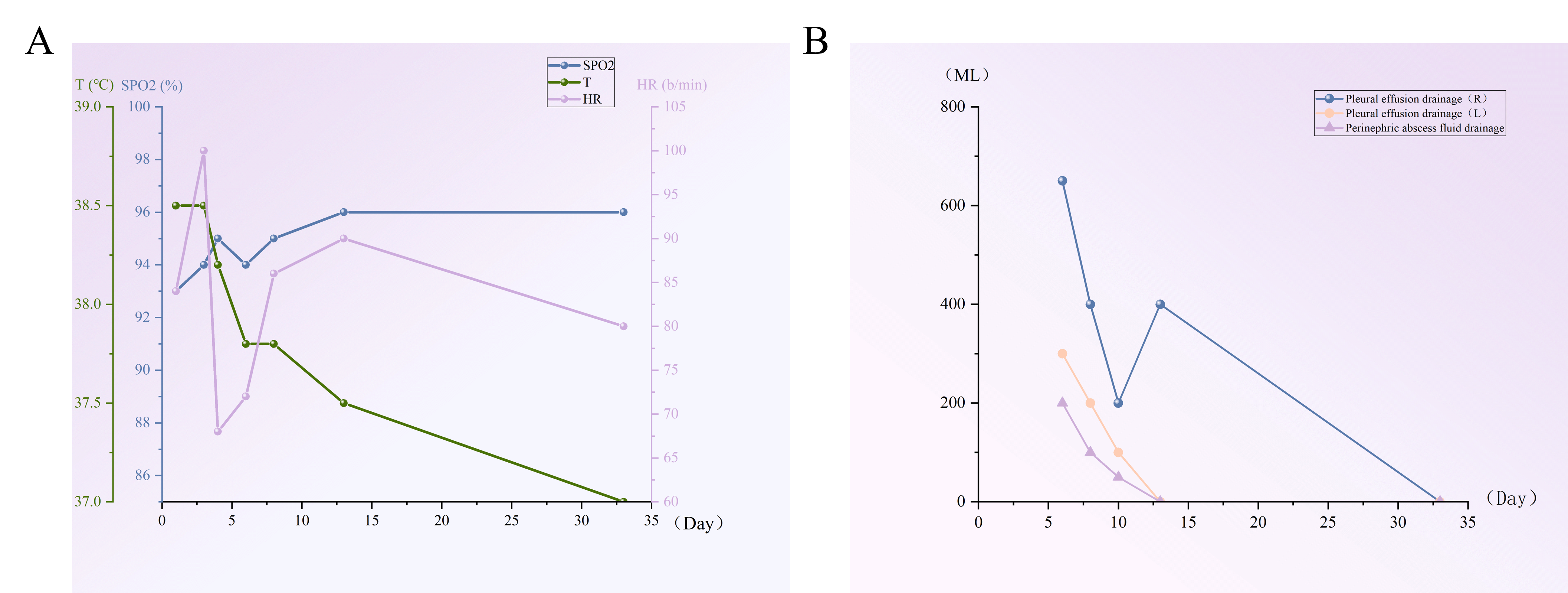

Supplement: Supplementary Figure 3 — Nursing record. (A) Shows the patient’s temperature, heart rate, and blood oxygen level changes at key time points during the illness. (B) Shows the changes in pleural effusion drainage and perinephric abscess drainage at key time points during the illness. [file Image3.tif]
